# Development and validation of a nomogram to predict the risk of surgical site infection within 1 month after transforaminal lumbar interbody fusion

**DOI:** 10.1186/s13018-023-03550-w

**Published:** 2023-02-14

**Authors:** Jiashu Lian, Yu Wang, Xin Yan, Guoting Xu, Mengxian Jia, Jiali Yang, Jinwei Ying, Honglin Teng

**Affiliations:** 1 Department of Orthopedics (Spine Surgery), The First Affiliated Hospital of Wenzhou Medical University, Wenzhou, 32500 Zhejiang China; 2 Department of Pediatric Allergy and Immunology, The Second Affiliated Hospital and Yuying Children’s Hospital of Wenzhou Medical University, Wenzhou, 325027 Zhejiang China

**Keywords:** Surgical site infection (SSI), Transforaminal lumbar interbody fusion (TLIF), Nomogram, Lumbar paraspinal muscles (LPM) fat infiltration, Surgery duration

## Abstract

**Objective:**

Surgical site infection (SSI), a common serious complication within 1 month after transforaminal lumbar interbody fusion (TLIF), usually leads to poor prognosis and even death. The objective of this study is to investigate the factors related to SSI within 1 month after TLIF. We have developed a dynamic nomogram to change treatment or prevent infection based on accurate predictions.

**Materials and methods:**

We retrospectively analyzed 383 patients who received TLIF at our institution from January 1, 2019, to June 30, 2022. The outcome variable in the current study was the occurrence of SSI within 1 month after surgery. Univariate logistic regression analysis was first performed to assess risk factors for SSI within 1 month after surgery, followed by inclusion of significant variables at *P* < 0.05 in multivariate logistic regression analysis. The independent risk variables were subsequently utilized to build a nomogram model. The consistency index (C-index), calibration curve and receiver operating characteristic curve were used to evaluate the performance of the model. And the decision curve analysis (DCA) was used to analyze the clinical value of the nomogram.

**Results:**

The multivariate logistic regression models further screened for three independent influences on the occurrence of SSI after TLIF, including lumbar paraspinal (multifidus and erector spinae) muscles (LPM) fat infiltration, diabetes and surgery duration. Based on the three independent factors, a nomogram prediction model was built. The area under the curve for the nomogram including these predictors was 0.929 in both the training and validation samples. Both the training and validation samples had high levels of agreement on the calibration curves, and the nomograms C-index was 0.929 and 0.955, respectively. DCA showed that if the threshold probability was less than 0.74, it was beneficial to use this nomograph to predict the risk of SSI after TLIF. In addition, the nomogram was converted to a web-based calculator that provides a graphical representation of the probability of SSI occurring within 1 month after TLIF.

**Conclusion:**

A nomogram including LPM fat infiltration, surgery duration and diabetes is a promising model for predicting the risk of SSI within 1 month after TLIF. This nomogram assists clinicians in stratifying patients, hence boosting decision-making based on evidence and personalizing the best appropriate treatment.

## Introduction

Lumbar degenerative disease (LDD), a chronic disease that gradually evolves into lumbar disk herniation, lumbar spinal stenosis or lumbar spondylolisthesis with age, leading to low back pain and lower limb sensory and motor dysfunction, has become one of the main causes of global human dysfunction and economic burden [1–3]. With LDD reaching a specific stage, surgical treatment becomes an effective method to alleviate the symptoms of patients. Transforaminal lumbar interbody fusion (TLIF) is one of the common surgical procedures for the treatment of LDD, which can significantly reduce the damage to muscles and nerves [4–6].

Surgical site infection (SSI) is a common serious complication within 1 month after TLIF, which can lead to nerve injury, sepsis and even death. Previous studies have shown that the incidence of SSI after TLIF is 2–8.5% [7], and more than 20% of patients readmitted within 30 days after surgery are caused by SSI [8, 9]. SSI not only increases the risk of revision surgery and prolongs the hospital stay, but also brings huge economic burden to patients, families and society. It also seriously affects the prognosis of patients, causing psychological burden and even death [3, 4]. Therefore, fully understanding the risk factors of SSI occurrence and integrating various related factors to develop a comprehensive prediction model to assess the risk of patients’ SSI will help spine surgeons reduce the incidence of SSI after TLIF. However, most of the previous studies on risk factors of SSI after spine surgery were mostly related to age, diabetes, obesity and the number of surgical segments. Few studies focused on the correlation between LPM fat infiltration and SSI after surgery.

Lumbar paraspinal (multifidus and erector spinae) muscles (LPM) are a muscle group including psoas multifidus and psoas erector spinalis, changing with age, including size reduction and increased fat infiltration [10]. The occurrence of LPM fat infiltration is due to the accumulation of a certain amount of fat tissue between LPM and vertebral lamina to replace the normal structure of muscle [11, 12]. Many previous studies have confirmed that fat infiltration is related to infection [13]. This may be because fat infiltration hinders the exposure of the surgical site, which increases the difficulty and duration of surgery [14]. It may also be that less blood vessels and tissue oxygenation in fat tissue increase the risk of tissue necrosis after wound closure [15]. It is also reported that fat infiltration increases the occurrence of SSI, which may be because it reduces the inherent stability of the lumbar spine [16, 17]. In addition, Sang et al. [18] have reported that weight distribution indicators such as lumbar multifidus muscle (LMM) may better predict the risk of SSI after lumbar surgery than BMI. However, the relationship between LPM fat infiltration and SSI after TLIF has not been reported. And whether the occurrence of SSI is affected by multiple factors including LPM fat infiltration has also not been reported.

Therefore, this study aims to develop an accurate and simple method to predict the incidence of SSI within 1 month after TLIF by evaluating a group of possible risk factors including LPM fat infiltration.

## Material and methods

### Patients and risk factors

The clinical data of 383 patients who received TLIF treatment in our institution from January 1, 2019, to June 30, 2022 were collected. The inclusion criteria were as follows: (1) patients diagnosed as LDD (including lumbar spinal stenosis, lumbar disk herniation or lumbar spondylolisthesis) according to clinical manifestations and radiological characteristics; (2) the symptoms were not relieved or aggravated after 3–6 months of conventional conservative treatment, which seriously affected the patient’s work and life; (3) patients with TLIF performed by the same group of surgeons; (4) first lumbar surgery; and (5) undergoing single-segment or dual-segment TLIF. Exclusion criteria were as follows: (1) patients who had undergone open surgery; (2) patients who had undergone lumbar surgery; (3) patients who lacked imaging data; (4) patients with preoperative infection, including lumbar vertebrae or other parts; (5) emergency surgery; and (6) patients used hormones during treatment or patients had other metabolic diseases (except diabetes and hypertension). The study was approved by the medical ethics committee of the author’s hospital.

Characteristics including demographics, radiologically identifiable factors and surgery-related information were collected from hospital medical records. Demographic and surgical-related information included age, gender, body mass index (BMI), height, weight, drinking, smoking, hypertension, diabetes, hyperlipidemia, occupation, education, maximum body temperature, the total volume of drainage, the last volume of drainage, the duration of drainage, lengths of admission, albumin, the highest CRP, the last CRP, the highest WBC count, the last WBC count, the highest ESR count, the last ESR count, subcutaneous fat thickness, urinary tract infections (UTIs), number of surgery levels, endosseous implants, surgery duration and postoperative prophylactic antibiotics. Radiologically identifiable factors included lung infection and LPM fat infiltration.

### SSI diagnostic criteria

The patients were rechecked one week after operation and followed up for at least one year (including 1, 3, 6 and 12 months after the operation). All complications (including the occurrence of SSI) were recorded by the available electronic medical system. The diagnosis of SSI was based on the incision observation recorded in the course of the disease (including redness, fever, pain, abscess and exudation in the surgical site), and MRI or histopathological examination and/or the diagnosis of the attending physician. The definition of SSI in this study is based on the CDC standard (Centers for Disease Control and Prevention) and is designated as acute spinal infection within 30 days after TLIF [19]. The SSI included shallow incision infection, deep incision infection and organ space infection occurred within 30 days after operation.

In addition, the final infection of all patients with SSI in this study was controlled. The diagnosis of infection controlled was based on the incision observation recorded in the course of the disease (including redness, fever, pain, abscess and exudation which were not observed in the surgical site), and MRI or histopathology and/or the diagnosis of the attending physician.

### Patient management

Cefazolin was the first choice for all patients 30 min before operation. The duration of antibiotic use and drainage tube placement after operation is shown in Tables 1 and 2. For patients with SSI, we would collect the patient’s tissue or exudate for bacterial culture and drug sensitivity test. After collection, we would give empirical anti infection treatment with linezolid injection. After that, after the results of bacterial culture and drug sensitivity were obtained, we would adjust the antibiotics to treat the infection. All patients would be told to leave the hospital after infection control.

**Table 1:** Patient characteristics of the training sample and the validation sample

Characteristics	Training sample	Validation sample	*P* value
Number^b^	288	95	
Age (years)^a^	61.75 ± 9.82	61.49 ± 9.15	0.624
BMI (kg/m^2^)^a^	24.68 ± 3.06	24.38 ± 2.9	0.581
Height (mm)^a^	162.16 ± 7.59	162.57 ± 7.31	0.429
Weight (kg)^a^	64.96 ± 9.63	64.64 ± 10.19	0.996
The total volume of drainage (mL)^a^	310.12 ± 133.13	300.86 ± 125.84	0.614
The last volume of drainage (mL)^a^	28.95 ± 18.56	28.65 ± 18.41	0.959
The duration of drainage (days)^a^	5.84 ± 1.1	5.8 ± 1	0.580
Lengths of admission (days)^a^	14.01 ± 4.24	13.91 ± 4.15	0.660
Albumin (g/L)^a^	34.39 ± 3.27	34.92 ± 3.23	0.251
The highest CRP (mg/L)^a^	38.95 ± 30.24	43.43 ± 38.28	0.653
The last CRP (mg/L)^a^	22.85 ± 22.8	23.65 ± 25.65	0.750
The highest WBC count (109/L)^a^	12.98 ± 3.41	13.4 ± 3.53	0.459
The last WBC count (109/L)^a^	8.71 ± 2.94	9.67 ± 8.11	0.575
The highest ESR count (mm/h)^a^	24.98 ± 18.29	26.29 ± 19.97	0.818
The last ESR count (mm/h)^a^	22.94 ± 17.08	25.17 ± 19.15	0.489
Subcutaneous fat thickness (mm)^a^	12.42 ± 5.94	12.74 ± 5.26	0.361
LPM fat infiltration (%)^a^	28.07 ± 11.14	29.04 ± 10.93	0.354
*Gender*^*b*^			0.492
Female	138 (47.9%)	41 (43.2%)	
Male	150 (52.1%)	54 (56.8%)	
*Drinking*^*b*^			0.606
No	242 (84%)	77 (81.1%)	
Yes	46 (16%)	18 (18.9%)	
*Smoking*^*b*^			1.000
No	235 (81.6%)	77 (81.1%)	
Yes	53 (18.4%)	18 (18.9%)	
*Hypertension*^*b*^			0.344
No	164 (56.9%)	60 (63.2%)	
Yes	124 (43.1%)	35 (36.8%)	
*Diabetes*^*b*^			0.061
No	210 (72.9%)	79 (83.2%)	
Yes	78 (27.1%)	16 (16.8%)	
*Hyperlipidemia*^*b*^			0.850
No	190 (66%)	61 (64.2%)	
Yes	98 (34%)	34 (35.8%)	
*UTIs*^*b*^			0.772
No	248 (86.1%)	80 (84.2%)	
Yes	40 (13.9%)	15 (15.8%)	
*Lung infections*^*b*^			0.209
No	279 (97.2%)	95 (100%)	
Yes	8 (2.8%)	0 (0%)	
*Number of surgery levels*^*b*^			0.859
1	252 (87.5%)	82 (86.3%)	
2	36 (12.5%)	13 (13.7%)	
*Endosseous implants*^*b*^			0.504
BMP-2	214 (74.3%)	67 (70.5%)	
WRIGHT	74(25.7%)	28(29.5%)	
*Occupation*^*b*^			0.146
Sedentary occupation	5 (1.7%)	1 (1.1%)	
Physical work	117 (40.6%)	49 (51.6%)	
Others	166 (57.6%)	45 (47.4%)	
*Education*^*b*^			0.873
Primary and secondary school	266 (92.4%)	89 (93.7%)	
High school	7 (2.4%)	1 (1.1%)	
College	15 (5.2%)	5 (5.3%)	
*Maximum body temperature (°C)*^*b*^			0.090
36.5–38	206 (71.5%)	78 (82.1%)	
38–39	76 (26.4%)	17 (17.9%)	
> 39	6 (2.1%)	0 (0%)	
Surgery duration (h)^b^	0.398		
< 3	120 (41.7%)	47 (49.5%)	
3–4	119 (41.3%)	33 (34.7%)	
≥ 4	49 (17%)	15 (15.8%)	
*Postoperative prophylactic antibiotics (days)*^*b*^			0.794
< 3	187 (64.9%)	64 (67.4%)	
3–7	64 (22.2%)	18 (18.9%)	
≥ 7	37 (12.8%)	13 (13.7%)	

*BMI* body mass index; *CRP* C-reactive protein; *WBC* white blood cell; *ESR* erythrocyte sedimentation rate; *LPM* lumbar paraspinal (multifidus and erector spinae) muscles; and *UTIs* urinary tract infections

^a^Mean ± SD

^b^Percentage (%)

**Table 2:** Baseline data on SSI and non-SSI of patients in the training cohort

Characteristics	All cases of training sample	Non-SSI	SSI	*P* value
Number	288	259	29	
Age (years)^a^	61.75 ± 9.82	61.44 ± 9.63	64.48 ± 11.24	0.114
BMI (kg/m^2^)^a^	24.68 ± 3.06	24.64 ± 3.00	25.00 ± 3.58	0.553
Height (mm)^a^	162.16 ± 7.59	162.10 ± 7.49	162.66 ± 8.54	0.712
Weight (kg)^a^	64.96 ± 9.63	64.82 ± 9.40	66.29 ± 11.64	0.435
The total volume of drainage (mL)^a^	310.12 ± 133.13	309.20 ± 133.21	318.31 ± 134.48	0.728
The last volume of drainage (mL)^a^	28.95 ± 18.56	28.29 ± 18.73	34.86 ± 16.08	0.071
The duration of drainage (days)^a^	5.84 ± 1.10	5.86 ± 1.08	5.59 ± 1.30	0.198
Lengths of admission (days)^a^	14.01 ± 4.24	13.96 ± 4.11	14.41 ± 5.35	0.587
Albumin (g/L)^a^	34.39 ± 3.27	34.40 ± 3.31	34.23 ± 2.97	0.787
The highest CRP (mg/L)^a^	38.95 ± 30.24	37.59 ± 28.49	51.12 ± 41.47	0.022
The last CRP (mg/L)^a^	22.85 ± 22.80	22.83 ± 22.69	23.06 ± 24.15	0.959
The highest WBC count (109/L)^a^	12.98 ± 3.41	12.93 ± 3.31	13.43 ± 4.24	0.456
The last WBC count (109/L)^a^	8.71 ± 2.94	8.71 ± 2.77	8.70 ± 4.26	0.986
The highest ESR count (mm/h)^a^	24.98 ± 18.29	24.31 ± 18.17	30.93 ± 18.58	0.064
The last ESR count (mm/h)^a^	22.94 ± 17.08	22.65 ± 17.18	25.52 ± 16.17	0.392
Subcutaneous fat thickness (mm)^a^	12.42 ± 5.94	12.08 ± 5.84	15.42 ± 6.13	0.004*
LPM fat infiltration (%)^a^	28.07 ± 11.14	26.90 ± 10.57	38.50 ± 10.75	< 0.001*
*Gender*^*b*^				0.348
Female	138 (47.9%)	127 (49.0%)	11 (37.9%)	
Male	150 (52.1%)	132 (51.0%)	18 (62.1%)	
*Drinking*^*b*^				0.643
No	242 (84.0%)	219 (84.6%)	23 (79.3%)	
Yes	46 (16.0%)	40 (15.4%)	6 (20.7%)	
Physical work	117 (40.6%)	107 (41.3%)	10 (34.5%)	
Others	166 (57.6%)	150 (57.9%)	16 (55.2%)	
*Education*^*b*^				0.618
Primary and secondary school	266 (92.4%)	239 (92.3%)	27 (93.1%)	
High school	7 (2.4%)	7 (2.7%)	0 (0.0%)	
College	15 (5.2%)	13 (5.0%)	2 (6.9%)	
*Maximum body temperature (°C)*^*b*^				0.247
36.5–38	206 (71.5%)	182 (70.3%)	24 (82.8%)	
38–39	76 (26.4%)	72 (27.8%)	4 (13.8%)	
> 39	6 (2.1%)	5 (1.9%)	1 (3.4%)	
*Surgery duration (h)*^*b*^				< 0.001*
< 3	120 (41.7%)	119 (45.9%)	1 (3.4%)	
3–4	119 (41.3%)	110 (42.5%)	9 (31.0%)	
≥ 4	49 (17.0%)	30 (11.6%)	19 (65.5%)	
*Postoperative prophylactic antibiotics (days)*^*b*^				0.873
< 3	187 (64.9%)	167 (64.5%)	20 (69.0%)	
3–7	64 (22.2%)	58 (22.4%)	6 (20.7%)	
≥ 7	37 (12.8%)	34 (13.1%)	3 (10.3%)	

*BMI* body mass index; *CRP* C-reactive protein; *WBC* white blood cell; *ESR* erythrocyte sedimentation rate; *LPM* lumbar paraspinal (multifidus and erector spinae) muscles; and UTIs: urinary tract infections

*P < 0.05

*Statistically significant

^a^Mean ± SD

^b^Percentage (%)

### Measurement of LPM fat infiltration

**Fig. 1:**
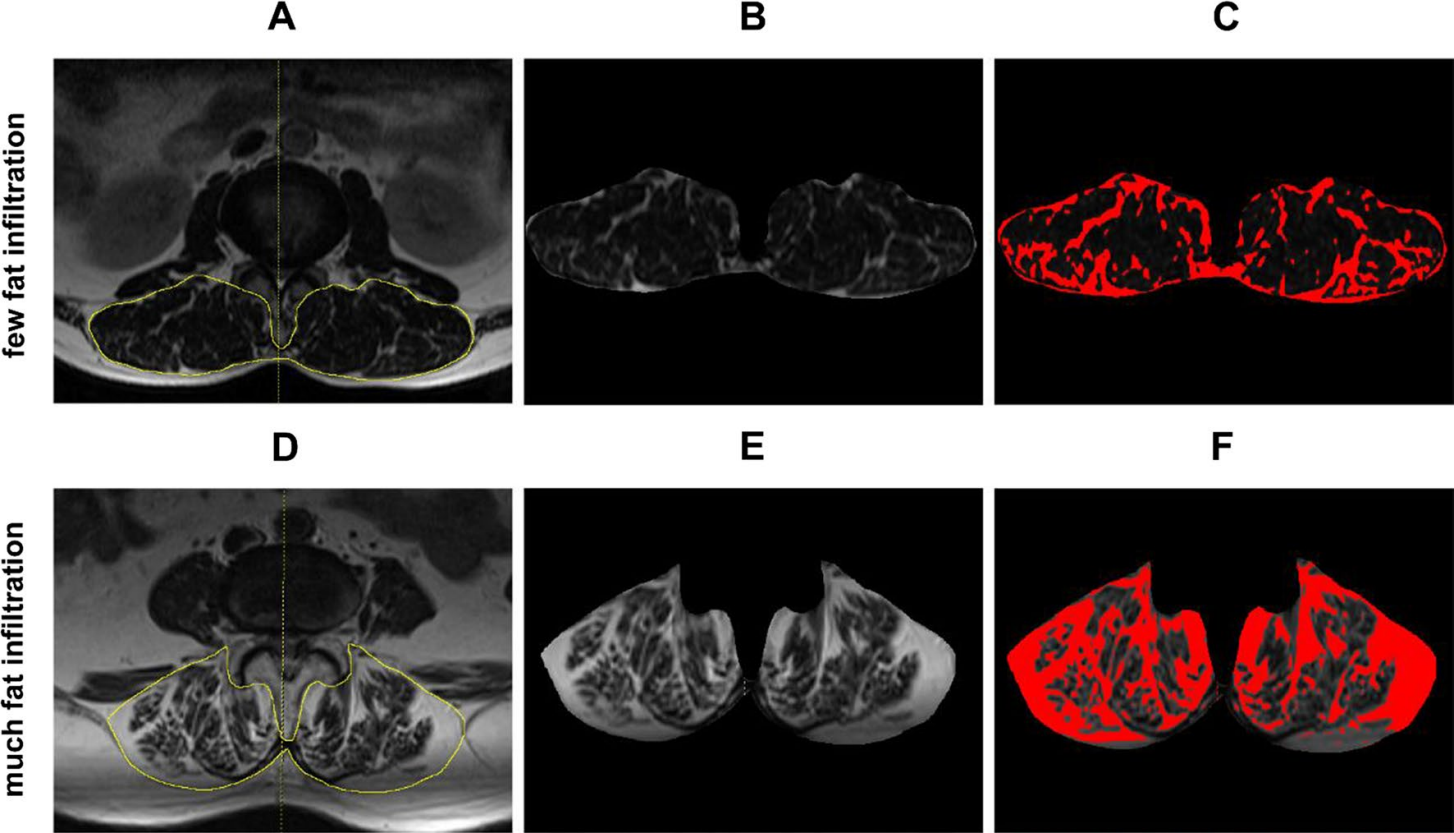
Cross section of the lumbar spine. T2WI MRI and calculation of the cross-sectional area of LPM and fat infiltration. **A**–**C** From the same patient with little fat infiltration; **D**–**F** From the same patient with much fat infiltration; **B**, **E** CSA of LPM on the same level; **C**, **F** CSA of fat tissue (red)

MRI (Siemens Magnetom Avanto1.5 T) of lumbosacral vertebrae came from 2 months before operation. Image J (Media Cybernetics, Bethesda, MD, USA) was used to obtain the cross-sectional area (CSA) of LPM (Fig. 1B, E) and fat tissue (Fig. 1C, F) at the surgical level. The formula for calculating the percentage of LPM fat infiltration was as follows (all measurements are performed at the surgical level) [18, 20]:

$${\text{LPM}}\;{\text{fat}}\;{\text{infiltration}}\left( \% \right) = {{{\text{CSA}}\;{\text{of}}\;{\text{fat}}\;{\text{tissue}}} \mathord{\left/ {\vphantom {{{\text{CSA}}\;{\text{of}}\;{\text{fat}}\;{\text{tissue}}} {{\text{CSA}}\;{\text{of}}\;{\text{LPM}}\;{\text{on}}\;{\text{the}}\;{\text{same}}\;{\text{level}}}}} \right. \kern-0pt} {{\text{CSA}}\;{\text{of}}\;{\text{LPM}}\;{\text{on}}\;{\text{the}}\;{\text{same}}\;{\text{level}}}} * 100$$

Finally, the accuracy of each measurement was manually verified by two orthopedic researchers and the measurement results showed no significant difference.

## Statistical analysis

R software (Version 4.1.2) was used for statistical analysis. The R packages used include car, survival, library, DynNom, shiny, plotly, compare, stargazer, glmnet, rms, ROCR and rmda [3, 21].

First, quantitative variables were tested by normality and homogeneity of variance, which were displayed as mean ± standard deviation (SD). Student’s t test was used for inter group comparison. Qualitative variables are described by frequency and percentage and compared by chi-square test. *P* < 0.05 was set as statistically significant. Univariate analysis and multivariate logistic regression analysis were conducted for each covariate to determine the predictors of SSI after TLIF.

Then, a nomogram model was established using variables with *P* less than 0.05 obtained from the single and multiple logistic regression. The nomogram was a model based on multiple logistic regression, which was compiled with R software.

Finally, the C-index and the ROC were used to evaluate the discrimination ability of nomogram on predicting the risk of SSI. The higher the C-index and the AUC of ROC curve, the better the discrimination. The calibration curves were constructed to evaluate the reliability of the nomogram. At the same time, the DCA was used to estimate the net benefits at various threshold probabilities in the cohort.

## Results

### Characteristics of patients

A total of 383 patients after TLIF were enrolled in this study and randomly divided into training group (288 patients) and validation group (95 patients). All their clinical data were carefully collected and sorted out (Table 1). There was no significant difference in the characteristics between the two groups from the table, which justified that they were reasonable as training groups and validation groups.

### Baseline data of the SSI and non-SSI groups

In our study, 29 patients in the training group and 11 patients in the validation group had postoperative SSI. Among the 40 patients with infection, 23 patients had positive results of bacterial culture in the secretion of the operation site, including 7 cases of staphylococcus epidermidis, 3 cases of enterococcus faecalis, 2 cases of enterococcus faecium, 2 cases of staphylococcus capitis, 2 cases of staphylococcus warneri, 1 case of staphylococcus aureus, human staphylococcus, staphylococcus coriolis urealyticus, enterobacter cloacae complex, Candida albicans, Acinetobacter baumannii and Candida guilliermondii.

In addition, we examined baseline data from 288 patients in the training cohort after TLIF through univariate analysis (Table 2). Data analysis displayed subcutaneous fat thickness, LPM fat infiltration, diabetes, UTIs, occupation and surgery duration were significant risk factors of SSI.

### Identification of independent risk factors

**Fig. 2:**
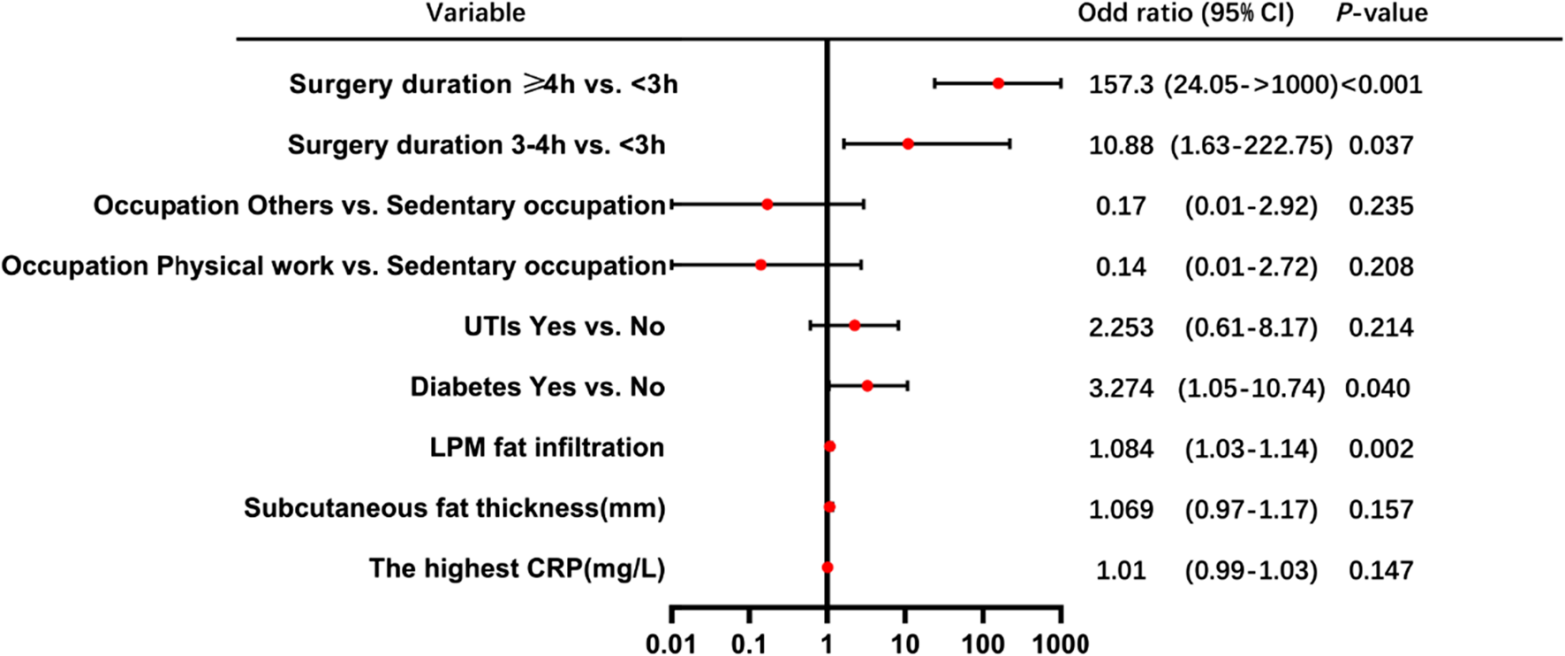
Multivariate logistic regression analysis of patients with SSI within 1 month after TLIF

In univariate regression analysis, the highest CRP, subcutaneous fat thickness, LPM fat infiltration, diabetes, UTIs, occupation and surgery duration showed statistically significant differences. These factors were further included in the multivariate logistic regression analysis (Table 3). Finally, LPM fat infiltration, diabetes and surgery duration were identified as independent risk factors of SSI within 1 month after TLIF (Fig. 2).

**Table 3:** Univariate and multivariate regression analysis for risk factors of SSI in the training cohort

Factors	Univariable analysis				Multivariable analysis			
	*β*	OR	95% CI	*P* value	*β*	OR	95% CI	*P* value
Age (years)	0.04	1.04	0.99–1.09	0.115				NI
BMI (kg/m^2^)	0.04	1.04	0.92–1.17	0.551				NI
Height (mm)	0.01	1.01	0.96–1.06	0.710				NI
Weight (kg)	0.02	1.02	0.98–1.06	0.434				NI
The total volume of drainage (mL)	0.01	1.00	0.99–1.00	0.727				NI
The last volume of drainage (mL)	0.02	1.02	1.00–1.04	0.074				NI
The duration of drainage (days)	− 0.23	0.79	0.55–1.13	0.198				NI
Lengths of admission (days)	0.02	1.02	0.93–1.11	0.586				NI
Albumin (g/L)	− 0.02	0.98	0.88–1.11	0.786				NI
The last CRP (mg/L)	0.01	1.00	0.98–1.02	0.959				NI
The highest WBC count (10^9^/L)	0.04	1.04	0.93–1.16	0.455				NI
The last WBC count (10^9^/L)	− 0.01	1.00	0.87–1.13	0.986				NI
The highest ESR count (mm/h)	0.02	1.02	1.00–1.03	0.069				NI
The last ESR count (mm/h)	0.01	1.01	0.99–1.03	0.392				NI
*Gender*								NI
Female	Ref	Ref	Ref	Ref				
Male	0.45	1.57	0.72–3.57	0.259				
*Drinking*								NI
No	Ref	Ref	Ref	Ref				
Yes	0.36	1.43	0.50–3.53	0.467				
*Smoking*								NI
No	Ref	Ref	Ref	Ref				
Yes	0.79	2.20	0.90–5.03	0.070				
> 7	− 0.31	0.74	0.17–2.30	0.637				
The highest CRP (mg/L)	0.01	1.01	1.00–1.02	0.025*	0.01	1.01	0.99–1.03	0.147
Subcutaneous fat thickness (mm)	0.09	1.09	1.03–1.16	0.005*	0.07	1.07	0.97–1.17	0.157
LPM fat infiltration (%)	0.08	1.08	1.05–1.12	< 0.001*	0.08	1.08	1.03–1.14	0.002*
*Diabetes*								
No	Ref	Ref	Ref	Ref				
Yes	1.53	4.60	2.10–10.38	< 0.001*	1.19	3.27	1.05–10.74	0.04*
UTIs								
No	Ref	Ref	Ref	Ref				
Yes	0.99	2.70	1.05–6.42	0.029*	0.81	2.25	0.61–8.17	0.214
*Occupation*								
Sedentary occupation	Ref	Ref	Ref	Ref				
Physical work	− 2.78	0.06	0.01–0.42	0.0043*	− 1.95	0.14	0.01–2.72	0.208
Others	− 2.64	0.07	0.01–0.46	0.0054*	− 1.80	0.17	0.01–2.92	0.235
*Surgery duration (h)*								
< 3	Ref	Ref	Ref	Ref				
3–4	2.28	9.74	1.79–181.01	0.03*	2.39	10.88	1.63–222.75	0.037*
≥ 4	4.32	75.37	14.74– > 1000	< 0.001*	5.06	157.34	24.05– > 1000	< 0.001*

*β* regression coefficient; *OR* odds ratio; *95% CI* 95% confidence interval; *NI* not included; and *Ref* reference

*P < 0.05

*Statistically significant

### Construction of the predicting SSI nomogram

**Fig. 3:**
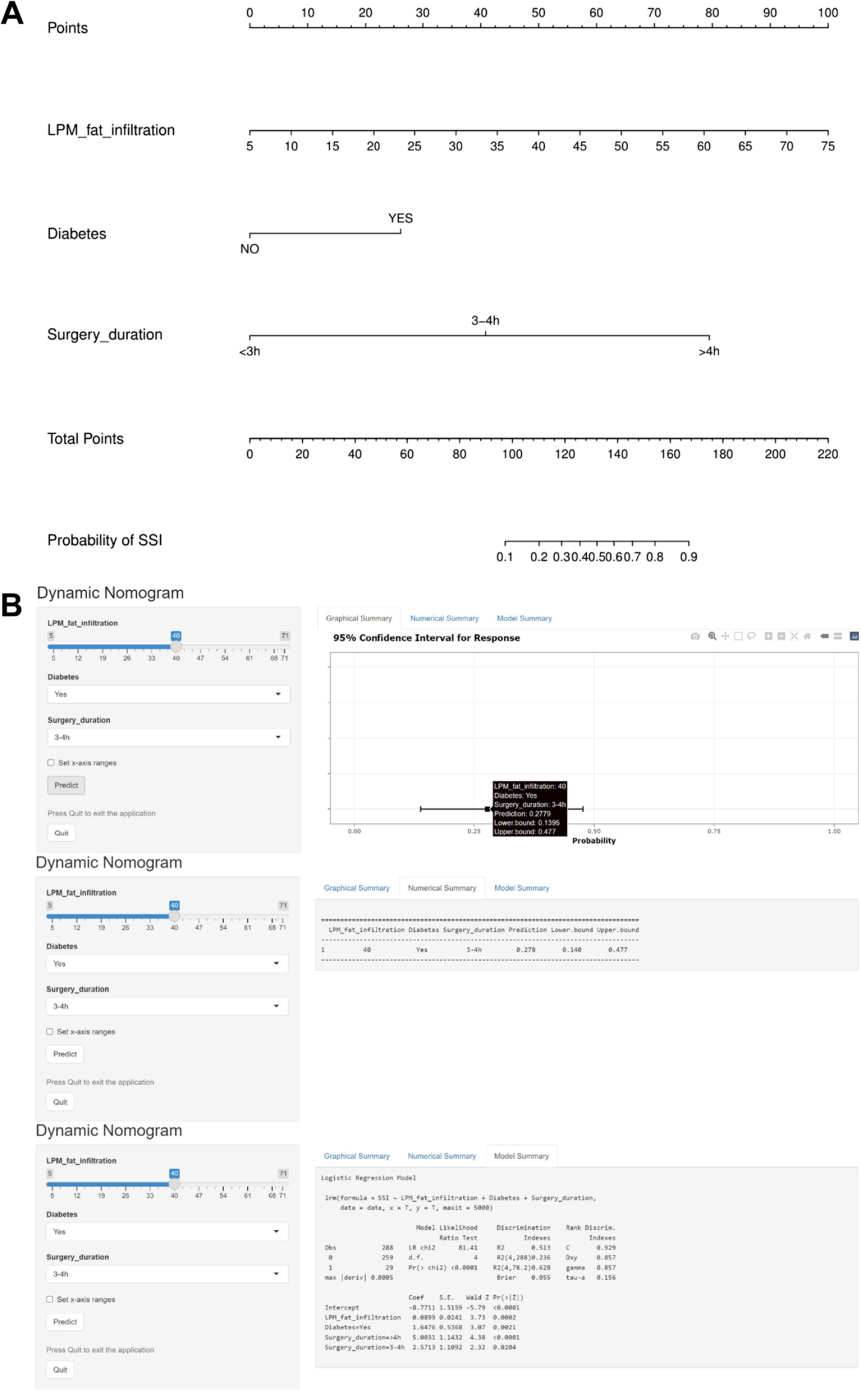
A nomogram predicting the risk of SSI within 1 month after TLIF. **A** The use of the nomogram is as follows: All variables of the patient (including LPM fat infiltration, surgery duration and diabetes) can obtain corresponding scores on the nomogram point axis. Add the corresponding scores of all variables to get the total score. The total score of each patient can be obtained through the corresponding relationship between the total score axis (score) and the SSI risk axis (%) to obtain the specific probability of SSI occurrence. **B** An online calculator converted from a nomogram is used to generate numerical predictions of the probability of SSI after TLIF (https://dynomogramrsci.shinyapps.io/DynNomSSI/)

Based on the results of multiple logistic regression analysis, LPM fat infiltration, diabetes and surgery duration were included in the prediction model to establish a nomogram, which visualized the regression analysis results (Fig. 3). The nomogram showed that LPM fat infiltration and surgery duration have great contribution to prediction. In addition, each factor in the nomogram was assigned a corresponding weighting point. The risk prediction of SSI within 1 month after TLIF was calculated by summing the weighting points of the three risk factors.

### Calibration and validation of the nomogram

**Fig. 4:**
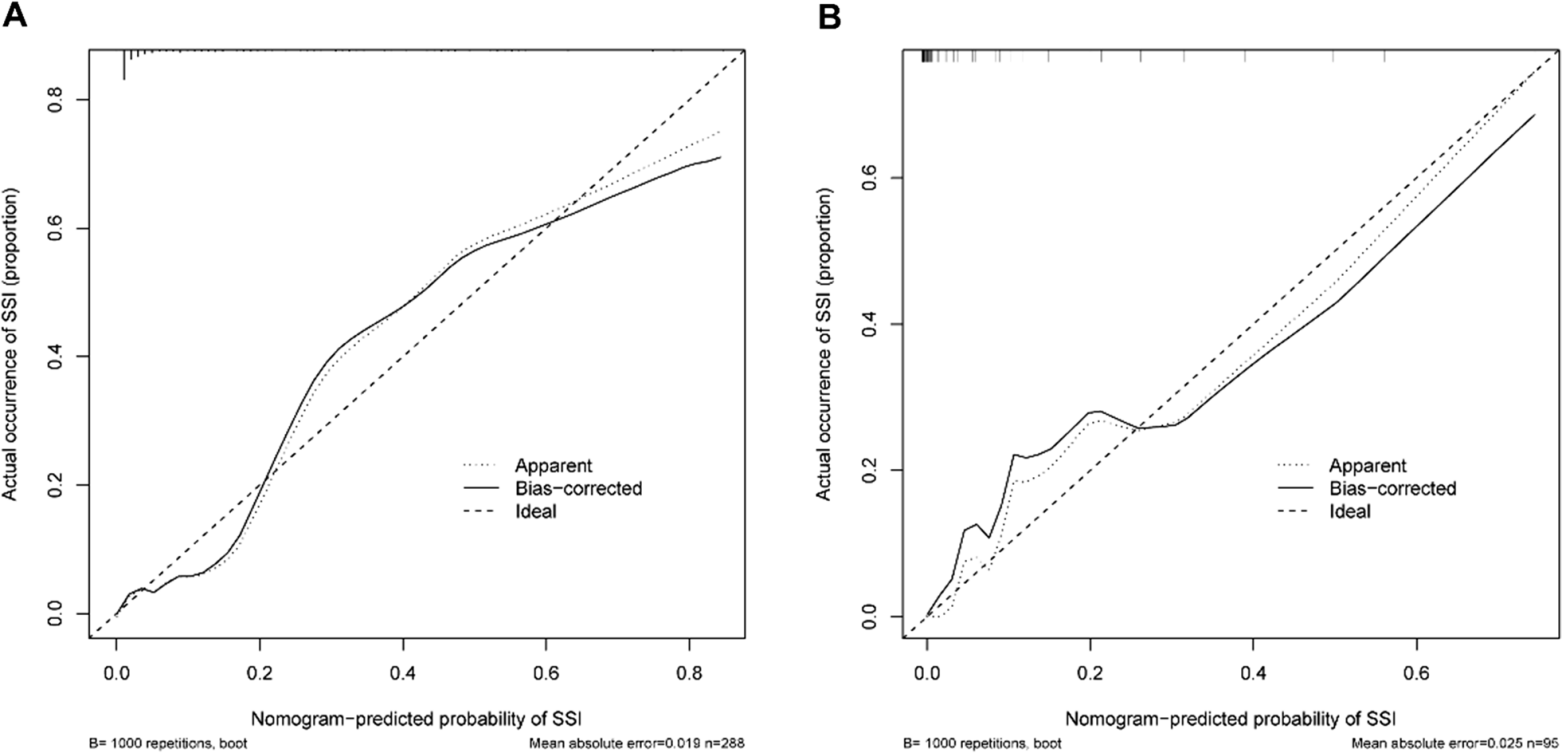
Calibration curves of the nomogram model in the training cohort (**A**) and validation cohort (**B**). Nomogram-predicted probability of SSI is plotted on the x-axis; actual probability of SSI is plotted on the y-axis. The diagonal dotted line represents the perfect prediction of the ideal model. The solid line represents the performance of the nomogram. The closer the fitting is to the diagonal dashed line, the more accurate the prediction is

**Fig. 5:**
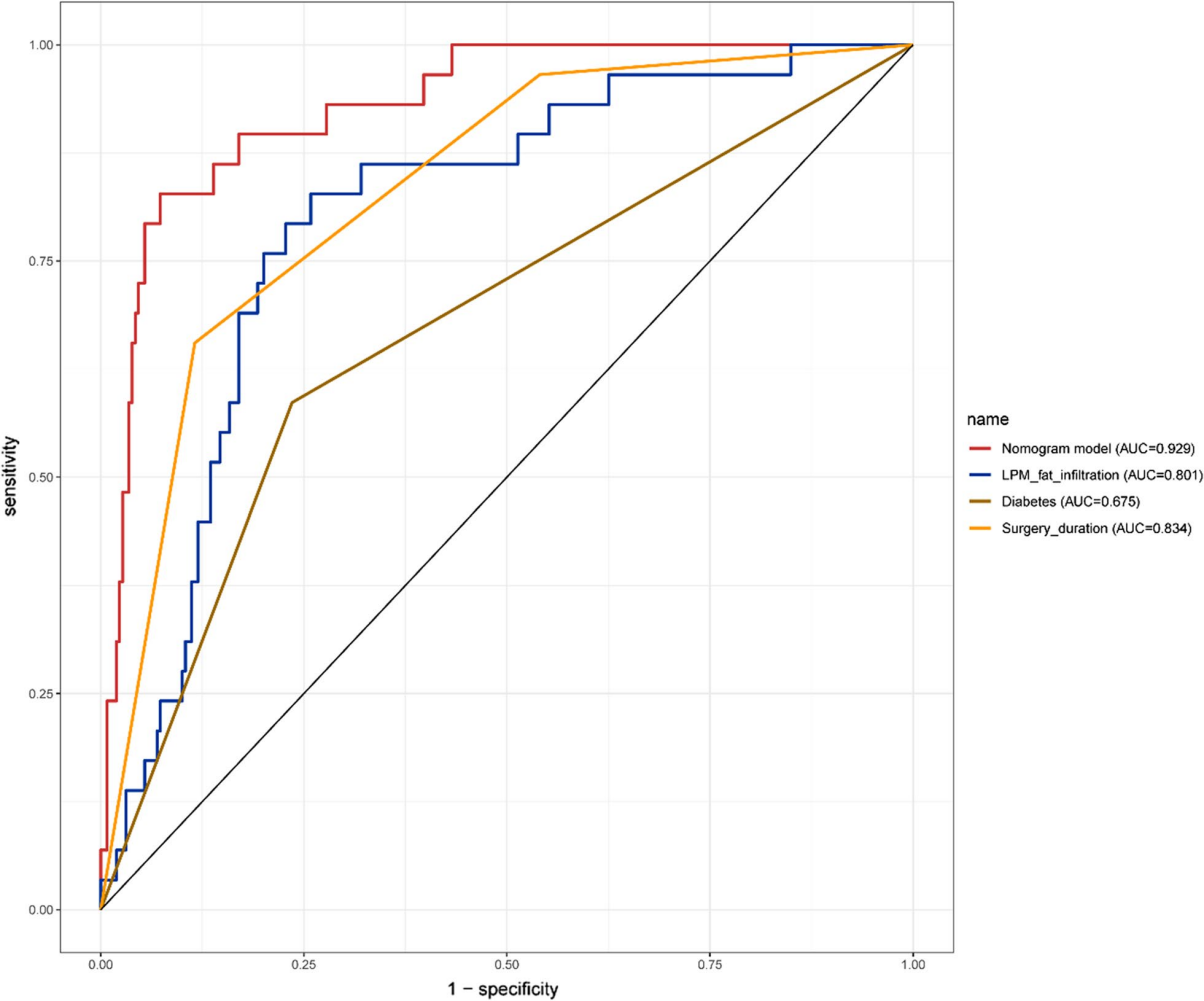
ROC curves of the training cohort. ROC curves of the predicting SSI nomogram showed that the AUC of the nomogram model, LPM fat infiltration, diabetes and surgery duration in the training cohort were 0.929, 0.801, 0.675 and 0.834, respectively

**Fig. 6:**
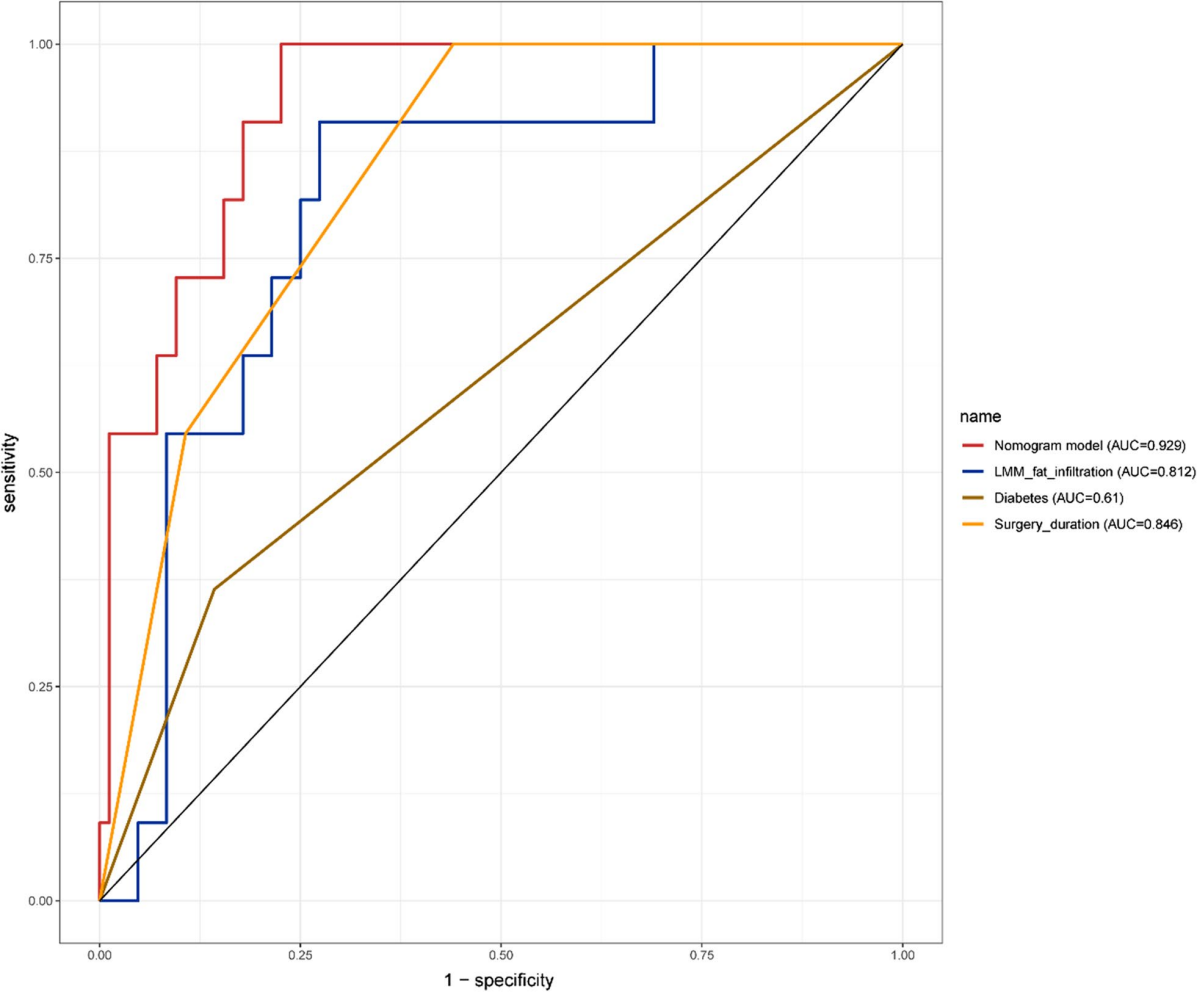
ROC curves of the validation cohort. ROC curves of the predicting SSI nomogram showed that the AUC of the nomogram model, LPM fat infiltration, diabetes and surgery duration in the validation cohort were 0.929, 0.812, 0.61 and 0.846, respectively

The calibration plots showed that the nomogram predictions were consistent with the actual observations of SSI risk within 1 month after TLIF in both the training (Fig. 4A) and validation (Fig. 4B) cohorts. And the nomograms C-index was 0.929 and 0.955, respectively. In addition, the ROC of the nomogram model used to predict the risk of SSI within 1 month after TLIF showed good discrimination (Figs. 5 and 6). The AUC of the nomogram model, LPM fat infiltration, diabetes and surgery duration in the training cohort was 0.929, 0.801, 0.675 and 0.834, respectively, while the AUC in the validation cohort was 0.929, 0.812, 0.61 and 0.846, respectively. Whether in the training cohort or the verification cohort, LPM fat infiltration and survey duration showed the great value of the nomogram.

### Predictive performance of nomogram

**Fig. 7:**
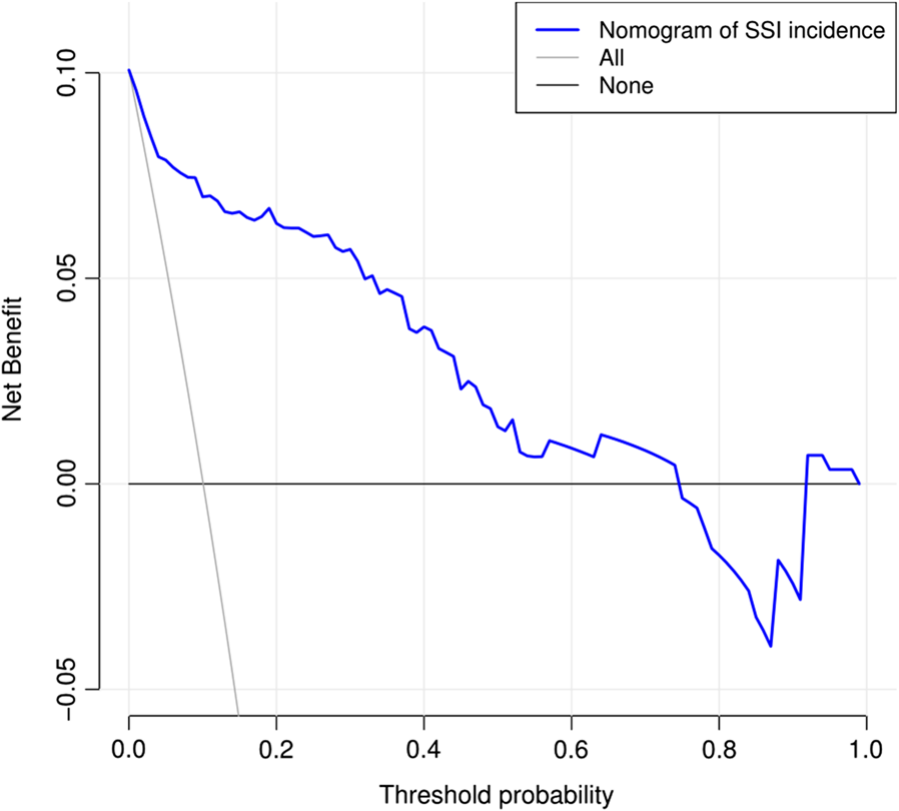
DCA of the nomogram model predicting SSI after TLIF. The solid blue line indicates the risk of SSI after TLIF predicted by the nomogram. The thin solid line indicates the assumption that SSI after TLIF is assumed to have occurred in all patients. The thick solid line indicates the hypothesis that no patients had SSI after TLIF. The decision curve demonstrated that using this nomogram in the current study to predict the risk of SSI after TLIF adds more benefit than either the intervention-all-patients scheme or the intervention-none scheme. This is the case if the threshold probability is less than 0.74. The x-axis shows the threshold probability. The y-axis represents the net benefit

DCA was conducted to assess the clinical significance of the predictive model, where showed the standardized net income and high-risk threshold (Fig. 7).

## Discussion

With the rise of the field of translational medicine, nomograms, as the important means in this field, have been pursued by more and more clinicians. Nomograms convert simple data into clinical prediction model through mathematical modeling, which greatly help clinicians to make more accurate judgments on corresponding diseases and formulate better treatment plans. However, the study of nomograms on SSI after TLIF is mainly aimed at patients with diabetes at present and cannot be widely applied to all patients. Therefore, we propose a nomogram for the first time to predict the risk of SSI after TLIF based on the degree of LDM fat infiltration.

SSI within 1 month after TLIF is one of the most common and serious complications. The occurrence of SSI not only seriously affected the prognosis of patients, but also caused a huge economic burden. Therefore, clinicians have never been negligent in improving surgical techniques, postoperative care and the use of perioperative preventive antibiotics [3, 4, 7]. At the same time, the researches on risk factors of SSI are also increasingly in-depth. Most of the previous studies on risk factors are related to age, diabetes, subcutaneous fat thickness, number of surgical segments, and so on [22]. However, it has been reported recently that fat infiltration of LMM may be a new risk factor for SSI [18].

LPM is the muscle group around the lumbar spine including LMM. Similar to lumbar degeneration, LPM decreases in size and increases in fat infiltration with age, which affect the stability of the lumbar spine [10, 18]. Many studies have shown that fat infiltration is closely related to infection [13], while most studies on LPM fat infiltration are related to low back pain [11, 12], and few articles have reported the correlation between LPM fat infiltration and SSI after lumbar surgery.

Considering that most of the previous studies are single factor analysis results, there is a lack of focus on multi factors risk assessment of patients. Our research is based on multiple logistic regression analysis. First, we preliminarily select 32 potential risk variables for research according to the data previously reported.

Secondly, univariate regression analysis is used to identify seven independent risk factors related to SSI, including the highest CRP, subcutaneous fat thickness, LPM fat infiltration, diabetes, UTIs, occupation and surgery duration. Finally, we select only three of the seven independent risk factors through multivariable regression analysis, including LPM fat infiltration, surgery duration and diabetes, which are easily obtained in routine clinical practice. The good calibration curve, high C-index and AUC values of the nomogram show that the method is feasible and has accurate prediction ability. In addition, the validation cohort further confirms that the nomogram can be widely used to predict the risk of SSI within 1 month after TLIF. DCA shows that when the threshold probability is less than 0.74, it is clinically valuable to use nomogram to predict the risk of SSI within 1 month after TLIF.

In our study, we find that the increased risk of SSI is associated with LPM fat infiltration. Previous articles have reported that it may be difficult to fully expose the operation site due to fat shielding, which also increases the difficulty and duration of the operation [14]. In addition, adipose tissue has fewer blood vessels and lower tissue oxygenation than muscle tissue, which may increase the risk of tissue necrosis and dead space development after wound closure and promote the occurrence of infection [15]. In addition, LPM fat infiltration reduces the inherent stability of the lumbar spine, which may also be the cause of increased SSI [16, 17]. Previous studies have reported that LMM fat infiltration is a new spine specific risk factor for SSI after lumbar surgery. In our study, we find that LPM has varying degrees of fat infiltration, not just LMM. Therefore, our study chose LPM fat infiltration as one of the risk factors affecting the occurrence of SSI. On this basis, we combine LPM fat infiltration with surgery duration and diabetes to comprehensively predict the risk of SSI after operation through multiple factor logistic regression analysis. Therefore, before LDD surgery, clinicians should pay more attention to the degree of LPM fat infiltration of patients and make a better surgical plan, including the determination of surgical methods and surgical approaches.

We also find that the increased risk of SSI is related to the surgery duration, which also corresponds to our previous analysis of the possible causes of LPM. In our study, we divide the surgery duration into three levels: less than 3 h, 3–4 h and more than or equal to 4 h. And we find that the longer the surgery duration, the higher the risk of SSI. According to our analysis, the longer the operation area is exposed, the easier it is to contact the pathogenic bacteria in the environment. Therefore, this is more consistent with our above views on the formulation of a good preoperative surgical plan, including difficulties that may arise during the operation and remedial measures.

In addition, we find that the increased risk of SSI is associated with diabetes. Most previous studies show that there is no significant difference between whether diabetes patients receive insulin treatment before surgery and the occurrence of SSI [23]. However, Liu et al. [3] recently report that insulin injection can reduce the incidence of SSI after surgery. We think that the stability of blood glucose is a prerequisite, and considering the reasons for fasting before surgery, the use of insulin may be a better choice.

This study shows that predictive models can help clinicians make evidence-based decisions to prevent SSI after TLIF. However, it is worth noting some limitations in this study. First of all, the limited sample size may weaken the statistical analysis of some risk factors and result in deviation. Secondly, only 3 risk factors are selected in this study, but previous studies have reported that other risk factors may be related to SSI, including age, number of surgery levels, postoperative prophylactic antibiotics, etc. In addition, our study is limited to 1 month after TLIF, which is based on the analysis of common cases in our medical institution and previous literature studies. For those patients with late infection, we will further improve in the follow-up study, including comparing the differences between early infection and late infection, so as to provide more perfect and targeted basis for clinical workers in formulating postoperative infection management plans.

## Conclusion

In summary, our research has successfully constructed a nomogram that can predict the risk of SSI within 1 month after TLIF. The nomogram established according to LPM fat infiltration, surgery duration and diabetes can help clinicians to stratify patients, thus promoting evidence-based decision-making and personalizing the best treatment plan. However, the reliability and applicability of this model need to be verified by more patient data and other medical institutions in the future.

## Data Availability

The data sets used and analyzed during the current study are available from the corresponding author on reasonable request.
